# The resemblance and disparity of gene expression in dormant and non-dormant seeds and crown buds of leafy spurge (*Euphorbia esula*)

**DOI:** 10.1186/s12870-014-0216-4

**Published:** 2014-08-12

**Authors:** Wun S Chao, Münevver Doğramaci, James V Anderson, Michael E Foley, David P Horvath

**Affiliations:** USDA-Agricultural Research Service, Biosciences Research Lab, Sunflower and Plant Biology Research Unit, 1605 Albrecht Boulevard N, Fargo, ND 58102 USA

**Keywords:** Leafy spurge, Bud dormancy, Seed dormancy, Gene expression, Hormones, Transcription factors

## Abstract

**Background:**

Leafy spurge (*Euphorbia esula* L.) is a herbaceous perennial weed and dormancy in both buds and seeds is an important survival mechanism. Bud dormancy in leafy spurge exhibits three well-defined phases of para-, endo- and ecodormancy; however, seed dormancy for leafy spurge is classified as physiological dormancy that requires after-ripening and alternating temperature for maximal germination. Overlaps in transcriptome profiles between different phases of bud and seed dormancy have not been determined. Thus, we compared various phases of dormancy between seeds and buds to identify common genes and molecular processes, which should provide new insights about common regulators of dormancy.

**Results:**

Cluster analysis of expression profiles for 201 selected genes indicated bud and seed samples clustered separately. Direct comparisons between buds and seeds are additionally complicated since seeds incubated at a constant temperature of 20°C for 21 days (21d C) could be considered paradormant (Para) because seeds may be inhibited by endosperm-generated signals, or ecodormant (Eco) because seeds germinate after being subjected to alternating temperature of 20:30°C. Since direct comparisons in gene expression between buds and seeds were problematic, we instead examined commonalities in differentially-expressed genes associated with different phases of dormancy. Comparison between buds and seeds (‘Para to Endo buds’ and ‘21d C to 1d C seeds’), using endodormant buds (Endo) and dormant seeds (1d C) as common baselines, identified transcripts associated with cell cycle (*HisH4*), stress response/transcription factors (*ICE2*, *ERFB4/ABR1*), ABA and auxin response (*ABA1*, *ARF1*, *IAA7*, *TFL1*), carbohydrate/protein degradation (*GAPDH_1*), and transport (*ABCB2*). Comparison of transcript abundance for the ‘Eco to Endo buds’ and ‘21d C to 1d C seeds’ identified transcripts associated with ABA response (*ATEM6*), auxin response (*ARF1*), and cell cycle (*HisH4*). These results indicate that the physiological state of 21d C seeds is more analogous to paradormant buds than that of ecodormant buds.

**Conclusion:**

Combined results indicate that common molecular mechanisms associated with dormancy transitions of buds and seeds involve processes associated with ABA and auxin signaling and transport, cell cycle, and AP2/ERF transcription factors or their up-stream regulators.

**Electronic supplementary material:**

The online version of this article (doi:10.1186/s12870-014-0216-4) contains supplementary material, which is available to authorized users.

## Background

Leafy spurge (*Euphorbia esula* L.) is considered an invasive perennial weed in the Upper Great Plains of North America and has been reported to cause significant economic losses [1]. Vegetative reproduction from an abundance of underground adventitious buds (often referred to as crown and root buds) and sexual reproduction through seeds allow leafy spurge to persist and spread. Dormancy in both buds and seeds is an important survival mechanism for leafy spurge and many other invasive perennial weeds. In leafy spurge, seed dormancy ensures distribution of germination in time and space, whereas bud dormancy inhibits underground adventitious buds from initiating new vegetative growth.

Dormancy classifications are different between bud and seed. In seeds, dormancy is defined as a developmental state in which germination fails under favorable environmental conditions [2]. Seed dormancy is also determined by both morphological and physiological properties [3–5]. Seed dormancy for leafy spurge is classified as physiological dormancy, which varies between populations from little or no dormancy to moderate periods of dormancy [6,7]. Physiological dormancy in leafy spurge generally can be released by cold or warm stratification. However, dormant leafy spurge seeds do not germinate at constant temperatures of 20°C or 30°C, but imbibing seeds for 21 days at constant temperature (20°C) followed by an alternating temperature (20:30°C) treatment increases germination to over 60% in 10 days [8].

Bud dormancy is subdivided into the three well-defined phases of para-, endo-, and eco-dormancy. Paradormancy (Para) is growth cessation controlled by physiological factors external to the affected structure, endodormacy (Endo) is growth cessation controlled by internal physiological factors, and ecodormancy is growth cessation controlled by external environmental factors [9]. Paradormancy in leafy spurge inhibits buds from developing into new shoots through signals such as auxin and sugars generated from the actively growing aerial portion of the plant [10–12], whereas endodormancy is triggered by cold temperature and short photoperiods in autumn [13–15]. Endodormancy is released, and ecodormancy (Eco) is maintained, by extended cold.

Seed and bud dormancy appears to involve similar physiological processes as both require abscisic acid (ABA) to induce dormancy and gibberellins (GA) to break dormancy, and both accumulate similar reserve proteins and lipids during dormancy [16,17]. Chilling has also been reported to break dormancy in seeds and buds of some species [18,19]. It has been suggested that some common mechanisms may regulate both seed and bud dormancy [20]. We also hypothesized that common mechanisms likely overlap in regulation of dormancy in buds and seeds of leafy spurge.

Although phenotypic analysis of mutants or transgenic plants is a primary strategy to understand the function/role of plant regulators (genes or hormones), the strategy is not often suitable for plants difficult to perform these alterations as in the case of leafy spurge. Comparative transcriptome analysis on buds and seeds is a good complement and would assist in the identification of conserved cell processes and important expression programs that are difficult to achieve using mutagenesis or transgenic approaches. Leafy spurge is a model perennial to investigate both seed and bud dormancy [12,15,21,22], and these investigations have identified a subset of genes involved in regulation of growth and development. Thus, in this study, the objectives are to identify commonalities in differentially-expressed genes, common trends in gene expression, and general molecular mechanisms during bud and seed dormancy and its release. Identification of common molecular processes regulating dormancy in seeds and buds in leafy spurge should provide new insights about common regulators of dormancy induction and release.

## Results and discussion

### Quantitative real time - polymerase chain reaction (qRT-PCR)

This study compared various phases of dormancy between crown buds (designated as “buds” throughout the text) and seeds using physiologically analogous dormancy conditions based on information obtained through previous dormancy studies in leafy spurge buds and seeds. Two hundred and one leafy spurge homologs of *Arabidopsis* genes involved in growth, hormone, light, and temperature response/regulation were selected for analysis (Additional file 1: Table S1). Gene expression by qRT-PCR was examined using total RNA prepared from seed and bud samples. Although all 201 primer pairs were designed based on sequences obtained from a leafy spurge EST-database (for details, see M & M), the possibility exists for different paralogues and alleles of target genes being amplified by a given primer pair. For this reason, we examined all the amplicons in the form of melting point curves (melting point temperatures; Tm) and visualization by gel electrophoresis (see Additional file 2: Table S2) for each of our primer pairs. The results indicated that the majority of these amplicons are unique. Among 201 genes, only 15 showed > one melting point curve (with 2 Tm values). However, our results showed that melting curve analysis alone was insufficient to recognize all specific/nonspecific amplification; for example, *COP1* (Primer # MD-041, lane 62) was observed as a single amplicon in agarose gel, but dissociation analysis generated two melting point curves (see melting point curves of these two genes in Additional file 2: Table S2). Since other factors such as G/C rich, amplicon misalignment in A/T rich regions, and secondary structure in the amplicon region can cause melting of DNA molecules in multiple phases [23], gel visualization of DNA bands is needed to accurately diagnose the number and size of amplicons.

Interestingly, some of the non-unique amplicons showed a migration in amplicon sizes under different phases of dormancy or in different organs; for example, *DREB A-1*/*DREB1D* (Primer # 598, agarose gel lane 44) was expressed as a single amplicon in all samples except endodormant buds (Endo), and *ATSR1* (Primer # 609, agarose gel lane 46) was expressed as a single amplicon in 1d C and 21d C seeds but as double amplicons in all other samples (see melting point curves of these two genes in Additional file 2: Table S2). Therefore even if the multiple products are amplified by a given primer pair, the differential accumulation of transcripts from a given gene family still indicate their response to physiological processes associated with comparable phases of dormancy.

### Cluster analysis

**Figure 1:**
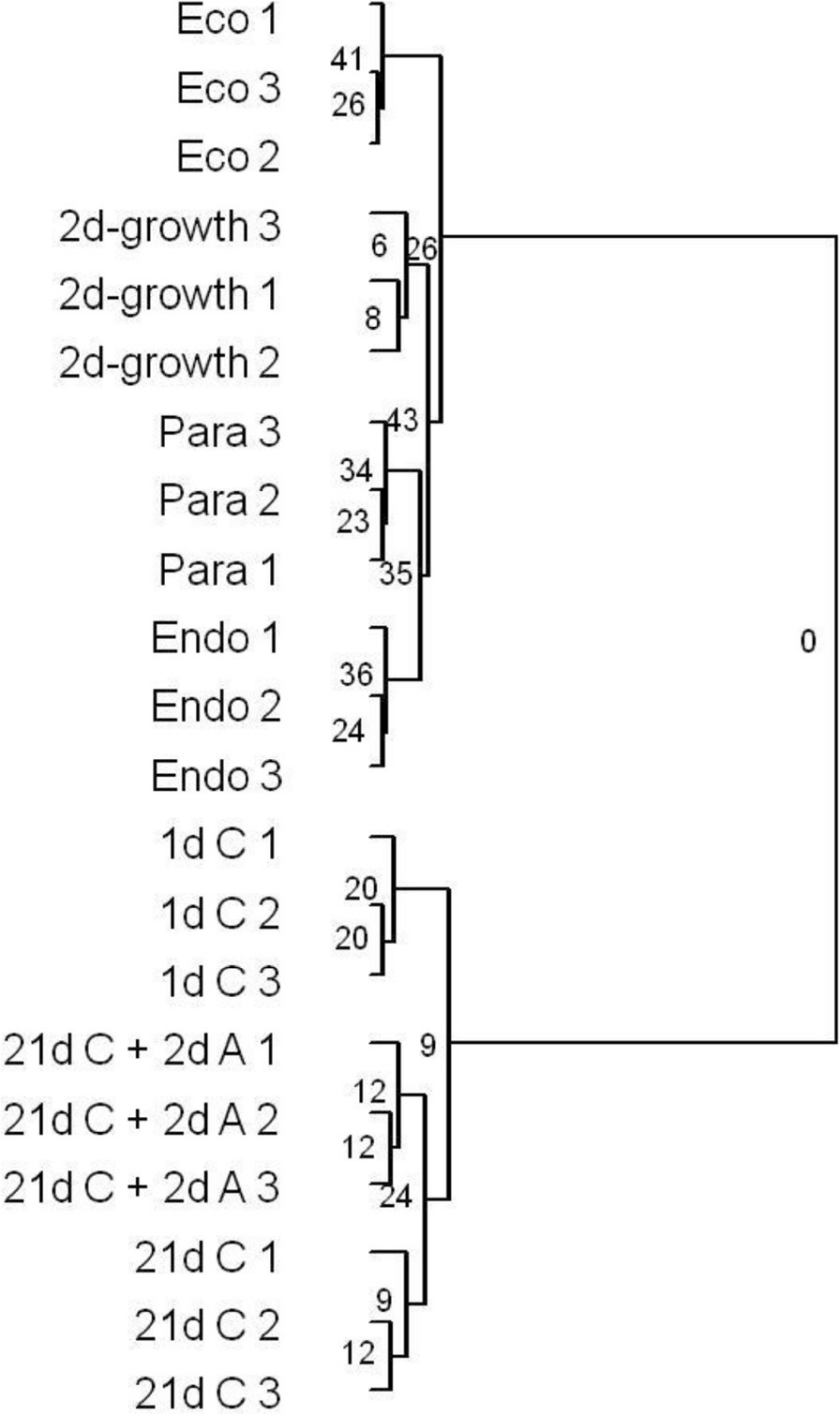
**Cluster analysis of bud and seed expression data.** Abbreviations for bud (Para, Endo, Eco, and 2d-growth) and seed (1d C, 21d C, and 21d C + 2d A) statuses are defined in Figures 2 and 3.

**Figure 2:**
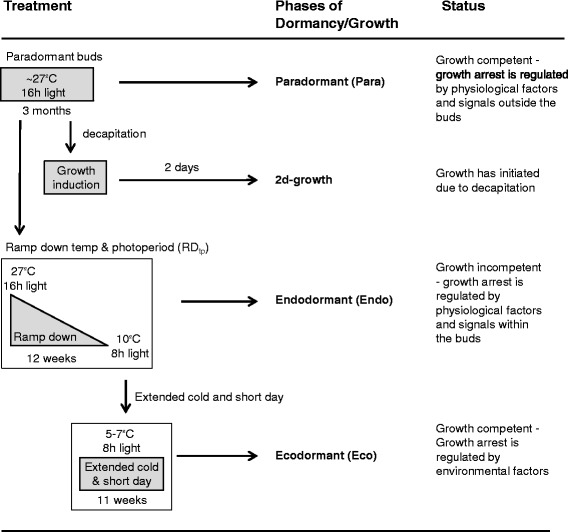
**Environmental treatments used and bud status for qRT-PCR analysis.**

**Figure 3:**
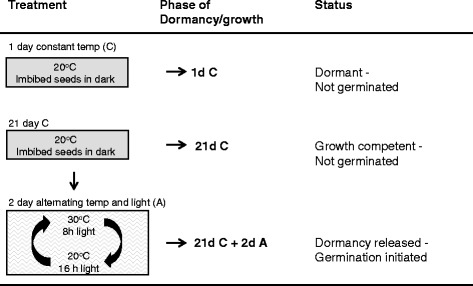
**Treatments abbreviations and seed status for qRT-PCR analysis.**

Cluster analysis on the expression profiles of 201 genes (Additional file 1: Table S1) indicated that buds and seeds fell into two main groups (Figure 1). One group contained all bud samples (Figure 2); Eco, Endo, Para, and 2d-growth (after paradormancy release). The second group contained all seed samples (Figure 3); 1d C (dormant), 21d C + 2d A (germinating), and 21d C (germination competent but inhibited by environmental or physiological signals). Even though buds and seeds clustered separately (Figure 1), it is possible that common physiological processes associated with dormancy states exist between them. For example, although 2d-growth and 21d C + 2d A both contained growing meristems, this similarity did not make these two samples cluster together.

These results suggest that substantial transcriptomic divergence may exist between buds and seeds, which could be due to differences in tissue types or other physiological, developmental, or environmental states. Consequently, direct comparison between buds and seeds was difficult. To overcome this barrier, we selected two common baselines to determine trends in differentially-expressed genes and identify common processes between analogous dormancy phases of buds and seeds. The endodormant phase was used as the baseline for buds, whereas 1d C (dormant) was used as the baseline for seeds.

### The physiological state of 21d C seeds is more analogous to paradormant buds than that of ecodormant buds

Seeds incubated for 1 day at the constant temperature of 20°C (1d C) will not germinate at optimal growth conditions; however, seeds incubated at a constant temperature of 20°C for 21 days (21d C) will germinate when subjected to alternating temperatures of 20:30°C [8] (see also Figure 3). Thus, the physiological state of 21d C seeds could be comparable to paradormant buds if seed germination was inhibited by endosperm-generated signals. In contrast, the physiological state of 21d C seeds could also be comparable to ecodormant buds if seed germination was inhibited by mechanisms such as a requirement for diurnal temperature variation. Neither endodormant buds nor 1d C seeds will germinate at optimal growth conditions and, for reasons mentioned above, they were used as common baselines for buds and seeds, respectively. We first determined differentially-regulated genes within buds (i.e., ‘Para to Endo’ or ‘Eco to Endo’) and seeds (i.e., ‘21d C to 1d C’) for the 201 genes by qRT-PCR (Additional file 1: Table S1). Transcript abundance for 48, 29, and 64 genes was significantly different (p < 0.1) in ‘Para to Endo’, ‘Eco to Endo’, and ‘21d C to 1d C’ comparisons, respectively (Additional file 3: Table S3). Common differentially-expressed genes were then identified based on the following comparisons: (1) paradormant buds vs. growth-competent seeds (‘Para to Endo’ vs. ‘21d C to 1d C’), and (2) ecodormant buds vs. growth-competent seeds (‘Eco to Endo’ vs. ‘21d C to 1d C’) (Tables 1 and 2).

**Table 1:** **Fold changes were represented by positive and negative fold numbers**

**Process**	**Gene**	**TAIR ID**	**Fold change**	**Fold change**
			**(‘Para to Endo’)**	**(‘21d C to 1d C’)**
**ABA**				
ABA biosynthesis	*ABA1*	At5g67030	−2.50*	−1.81*
**Auxin**				
ABC transporter	*ABCB2*	At4g25960	−2.74**	−2.92**
Auxin AUX/IAA	*IAA7*/*AXR2*	At3g23050	−4.98**	−2.78*
Auxin response	*ARF1*	At1g59750	−1.68*	−2.73**
**Cytokinin**				
Cytokinin catabolic process	*CKX5*	At1g75450	4.45*	−3.53*
**Gibberellic acid**				
GA response - receptor	*GID1B*	At3g63010	5.79**	−1.54*
**Ethylene**				
AP2/ERF TF	*ERF B-3/ERF1*	At4g17500	6.16*	−8.42**
	*ERF B-4/ABR1*	At5g64750	−5.21**	−16.0**
Ethylene response - receptor	*ETR2*	At3g23150	2.31*	−4.09**
**Miscellaneous**				
Carbohydrate/protein degradation	*GAPDH_1*	At1g13440	−5.39**	−5.18**
Cell cycle	*Histone H4*	At1g07660	−2.00**	−3.15*
Flowering	*TFL1*	At1g18100	−1.74*	−8.27*
Phosphorylation	*MKK9*	At1g73500	2.99*	−2.25*
Stress response	*ICE2*	At1g12860	−2.24**	−2.77**
	*LEA 4-5*	At5g06760	0.20**	−11.1**

Fold changes for buds were determined by comparing the gene expression of paradormant buds to endodormant buds (‘Para to Endo’), and fold changes for seeds were determined by comparing the gene expression of 21-day C seeds to 1-day C seeds (‘21d C to 1d C’). Common genes were then identified between buds and seeds. The *Arabidopsis* Information Resource (TAIR) IDs represent *Arabidopsis* genes used to annotate homologues of leafy spurge transcripts. Unpaired two-sample t-tests were performed; symbol “*” represents genes at a p-value < 0.1, and “**” represents genes at a p-value < 0.05.

**Table 2:** **Fold changes were represented by positive and negative fold numbers**

**Process**	**Gene**	**TAIR ID**	**Fold change**	**Fold change**
			**(‘Eco to Endo’)**	**(‘21d C to 1d C’)**
**ABA**				
ABA response	*ABI1*	At4g26080	1.46*	−4.54**
	*ATEM6*	At2g40170	−4.79*	−7.69**
**Auxin**				
Auxin response	*ARF1*	At1g59750	−1.68*	−2.73**
	*GH3.1*	At2g14960	1.84*	−2.78**
	*RUB1*	At1g31340	1.56*	−2.50**
Auxin AUX/IAA	*IAA16*	At3g04730	2.06*	−4.35*
Auxin transporter	*PILS7*	At5g65980	2.43*	−11.0**
**Cytokinin**				
Cytokinin catabolic process	*CKX5*	At1g75450	3.06*	−3.53*
**Miscellaneous**				
Cell cycle	*Histone H4*	At1g07660	−1.84**	−3.15*
Stress response	*LEA 4-5*	At5g06760	8.16*	−11.1**

Fold changes for buds were determined by comparing the gene expression of ecodormant buds to endodormant buds (‘Eco to Endo’), and fold changes for seeds were determined by comparing the gene expression of 21-day C seeds to 1-day C seeds (‘21d C to 1d C’). Common genes were then identified between buds and seeds. The *Arabidopsis* Information Resource (TAIR) IDs represent *Arabidopsis* genes used to annotate homologues of leafy spurge transcripts. Unpaired two-sample t-tests were performed; symbol “*” represents genes at a p-value < 0.1, and “**” represents genes at a p-value < 0.05.

Comparison of transcript expression profiles between ‘Para to Endo’ buds and ‘21d C to 1d C’ seeds identified 15 common differentially-expressed genes (Table 1). Some transcript changes were significant but not large in amplitude. Nine of these genes showed the same trend in expression pattern. These 9 transcripts are involved in ABA biosynthesis (*ABA1*), auxin transport or response (*ABCB2*, *IAA7*/*AXR2*, *ARF1*), ethylene response (*ERF B-4*/*ABR1*), carbohydrate/protein degradation (*GAPDH_1*), cell cycle (*Histone H4*), flowering (*TFL1*), and stress response (*ICE2*). Six showed an opposite trend in expression pattern and are involved in cytokinin catabolic process (*CKX5*), GA response (*GID1B*), ethylene response (*ERF B-3/ERF1*, *ETR2*), phosphorylation (*MKK9*), and stress response (*LEA 4–5*).

The ABA biosynthetic gene *ABA1* was among those showing the same trend in expression pattern. This gene was down-regulated in both paradormant buds and 21d C seeds relative to endodormant buds and 1d C seeds, respectively. *ABA1* encodes zeaxanthin epoxidase which plays a role in the epoxidation of zeaxanthin to antheraxanthin and all-trans-violaxanthin in the ABA biosynthetic pathway. *ABA1* expression was significantly lower in the ABA deficient mutant (*aba1*) than those in wild-type *Arabidopsis*; in addition, exogenous ABA application enhanced the expression of *ABA1* significantly [24]. Therefore, the down-regulation of *ABA1* could indicate that ABA synthesis was lower in paradormant buds and 21d C seeds relative to endodormant buds and 1d C seeds. Genes involved in auxin transport (*ABCB2*) and response (*IAA7*/*AXR2*, *ARF1*) were also down-regulated in paradormant buds and 21d C seeds. *ABCB2* encodes p-glycoprotein (PGP) and facilitates the cellular and long-distance transport of auxin [25]. Both *IAA7*/*AXR2* and *ARF1* are auxin-responsive genes. In general, the transcription factor ARF proteins bind to the promoters of auxin-responsive genes to activate or repress transcription. *IAA7*/*AXR2* encodes an Aux/IAA protein which is a transcriptional regulator that represses transcription controlled by ARF [26,27]. The down-regulation of *ABCB2*, *IAA7*/*AXR2*, and *ARF1* suggested that there may be lower auxin signaling in paradormant buds and 21d C seeds relative to their baseline.

Comparison of transcript expression profiles between ‘Eco to Endo’ buds and ‘21d C to 1d C’ seeds identified 10 common differentially-expressed genes (Table 2). Similar to ‘Para to Endo’ and ‘21d C to 1d C’ comparison, some of their transcript changes were not large in amplitude. Among the10 common genes, only three showed the same trend in expression pattern. These 3 transcripts are involved in ABA response (*ATEM6*), auxin response (*ARF1*), and cell cycle (*Histone H4*). Seven showed an opposite trend in expression pattern and are involved in ABA response (*ABI1*), auxin response or transport (*GH3.1 RUB1*, *IAA16*, *PILS7*), cytokinin catabolic process (*CKX5*), and stress response (*LEA 4–5*).

The ABA responsive gene *ATEM6* and auxin responsive gene *ARF1* exhibited a similar down-regulated trend in expression pattern in ecodormant buds and 21d C seeds relative to endodormant buds and 1d C seeds, respectively. *ATEM6* is ABA-inducible and is expressed primarily in the shoot apical meristem and provascular tissue [28]. *ATEM6* encodes a group 1 LEA protein which may contribute to cellular stability within the desiccated seed. The down-regulation of *ATEM6* and *ARF1* suggested that there may be lower ABA and auxin signaling in ecodormant buds and 21d C seeds. Though this may be true for 21d C seeds, such conclusion may not apply to ecodormant buds as other ABA responsive (*ABI1*) and auxin responsive (*GH3.1*, *RUB1*) genes were slightly up-regulated. Overall, based on the number of genes and their trend in gene expression, the physiological state of 21d C seeds is more analogous to paradormant buds than that of ecodormant buds.

### Growth initiation induced auxin response/transport and cell expansion processes in both buds and seeds

Growth-induced buds (Figure 2) were compared with germination-induced seeds (Figure 3) to identify analogous physiological responses during the initial phase of bud and seed growth. We first determined differentially-expressed genes within buds (i.e., ‘2d-growth to Endo’) and seeds (i.e., ‘21d C + 2d A to 1d C’) for the 201 genes (Additional file 1: Table S1). Transcript abundance for 23 and 35 genes was significantly different (p < 0.1) in ‘2d-growth to Endo’ and ‘21d C + 2d A to 1d C’ comparisons, respectively (Additional file 3: Table S3). Comparison of buds and seeds (i.e., ‘2d-growth to Endo’ vs. ‘21d C + 2d A to 1d C’) identified 6 common differentially-expressed genes (Table 3), of which 3 had the same trend in expression. These 3 transcripts are involved in auxin transport (*PID*, *PIN3*) and growth (*EXP6*). The other 3 showed an opposite trend in expression pattern and are involved in auxin transport (*PILS7*), cytokinin catabolism (*CKX5*), and amino acid biosynthesis (*SK1*).

**Table 3:** **Fold changes were represented by positive and negative fold numbers**

**Process**	**Gene**	**TAIR ID**	**Fold change**	**Fold change**
			**(‘2d-growth to Endo’)**	**(‘21d C + 2d A to 1d C’)**
**Auxin**				
Auxin transporter	*PID*	At2g34650	1.30*	2.96**
	*PILS7*	At5g65980	3.04*	−10.0**
	*PIN3*	At1g70940	2.01*	4.15*
**Cytokinin**				
Cytokinin catabolic process	*CKX5*	At1g75450	8.11*	−3.42**
**Miscellaneous**				
Amino acid biosynthesis	*SK1*	At2g21940	2.67**	−2.20*
Growth	*EXP6*	At2g28950	2.06*	19.0**

Fold changes for buds were determined by comparing the gene expression of 2d-growth buds to endodormant buds (‘2d-growth to Endo’), and fold changes for seeds were determined by comparing the gene expression of 21d C + 2d A seeds to 1d C seeds (‘21d C + 2d A to 1d C’). Common genes were then identified between buds and seeds. The *Arabidopsis* Information Resource (TAIR) IDs represent *Arabidopsis* genes used to annotate homologues of leafy spurge transcripts. Unpaired two-sample t-tests were performed; symbol “*” represents genes at a p-value < 0.1, and “**” represents genes at a p-value < 0.05.

Transcript of *PID* and *PIN3* were up-regulated in both 2d-growth buds and 21d C + 2d A seeds relative to endodormant buds and 1d C seeds, respectively. These two genes are involved in asymmetric auxin distribution for the gravitropic response [29]. In addition, transcript of *EXP6* was up-regulated in 2d-growth buds and 21d C + 2d A seeds. *EXP6* is involved in the modulation of cell wall extensibility [30] and leaf growth [31]. Given the roles of *PID*, *PIN3*, and *EXP6* in various aspects of growth, the up-regulation of these genes, not surprisingly, imply similar processes are involved in initial stages of growth in both buds and seeds.

### MAF3 displayed >10-fold transcript abundance at specific phases of dormancy/growth

Genes that had large changes in transcript abundance (>10-fold) may reflect specific roles during various phases of dormancy in buds and seeds. These genes are listed (in red) in Additional file 3: Table S3. A flowering gene, *MAF3*, was strongly up-regulated (773-fold) in ecodormant buds relative to endodormant buds (Additional file 3: Table S3, ‘Eco to Endo’), and was undetectable in paradormant and growth-induced buds. In contrast, it was down-regulated (−15-fold) in germinating relative to dormant seeds (Additional file 3: Table S3, ‘21d C + 2d A to 1d C’). In *Arabidopsis*, MAF3 is down-regulated by long-term cold and is involved in inhibiting flowering by directly repressing the expression of florigen *FT* [32]. However, *MAF3* expression in leafy spurge buds appears opposite based on what is observed for this gene in *Arabidopsis* [33]. The fact that *MAF3* expression is down-regulated during seed germination and is down-regulated in growing buds relative to ecodormant buds suggest perhaps that *MAF3* is a negative regulator of growth. In poplar, FT is a positive regulator of growth [34] and in *Arabidopsis*, MAF3 inhibits FT expression, our observation would be consistence with this hypothesis.

## Conclusion

We compared transcript profiles in buds and seeds. Direct comparisons of qRT-PCR results were impractical due to intrinsic differences between buds and seeds. Therefore, we utilized two common baselines, endodormant bud and dormant seed samples, to compare and determine differentially-expressed genes. Genes responsive to dormancy states were then identified by comparing those differentially-expressed genes in buds and seeds. This approach helped identify common processes related to similar physiological states in leafy spurge crown buds and seeds. Based on the number of common genes identified and those showing the same trend in expression pattern, we conclude that physiological relatedness in some phases of dormancy and growth does exist between buds and seeds. These identified genes can be used as molecular markers for specific dormancy phases in both buds and seeds. Transcriptome analysis identified potentially important molecular mechanisms involved in dormancy induction and release. Based on the combined results, common molecular mechanisms involved in dormancy transitions of buds and seeds likely involve processes associated with ABA and auxin signaling and transport, cell cycle, and *AP2/ERF* transcription factors or their up-stream regulators. However, transcript abundance may not reflect a direct association with protein level and activity. Therefore, direct protein or hormone measurement would corroborate current results.

## Methods

### Plant material and germination

Leafy spurge buds were prepared according to Doğramacı et al. [14,15] (Figure 2). Briefly, leafy spurge plants were propagated from the uniform biotype (1984-ND001) and maintained in a greenhouse as described by Anderson and Davis [35]. Prior to the start of each experiment, plants were acclimated in a Conviron growth chamber (Model PGR15) for 1 week at 27°C, 16:8 h light:dark photoperiod. Each experiment was replicated three times, and each replicate contained 30 plants. Six plants from each replicate were used to determine vegetative growth rate, and crown buds from the remaining 24 plants were collected for qRT-PCR studies. All samples were collected between 11:00 a.m. and 1:00 p.m. central standard time to avoid diurnal variation. To induce growth, paradormant plants were decapitated and grown for 2 days at 27°C, 16:8 h light:dark photoperiod. To induce endodormancy, paradormant plants were subjected to a ramp-down in temperature (27 → 10°C) and photoperiod (16 h → 8 h light) for 12 weeks (i.e., RD_tp_). To induce crown buds from endo- to ecodormancy, plants subjected to the RD_tp_ treatment were given extended cold treatment for 11 weeks at 5–7°C, under constant 8 h:16 h light:dark cycle. A set of paradormant plants was kept under constant temperature and photoperiod (27°C, 16 h light) as a control. Endodormant buds were used as the baseline for transcriptome comparisons.

Field-grown leafy spurge seeds were collected from Fargo, ND USA in 2006, 2007, and 2008. Seed harvesting, drying, fractionation, storage, surface disinfection, imbibition in water, and germination were previously described [7,8]. In this study, three germination treatments (Figure 3) were subjected to qRT-PCR analysis: I) 1d C: seeds imbibed for 1 d at the constant temperature of 20°C. 1d C seeds were used as the baseline for transcriptome comparisons; II) 21d C: seeds imbibed for 21 d at the constant temperature of 20°C. III) 21d C + 2d A: seeds imbibed for 21 d at 20°C followed by 2 d at the alternating temperature (20:30°C/16:8 h). Seeds were kept in the dark, except for short period of rating and harvesting seeds. The 2006, 2007, and 2008 seed samples served as the biological replicates.

### qRT-PCR

Primer pairs (20–24 nucleotides) were designed using Lasergene (DNASTAR, Inc., Madison, WI) sequence analysis software from 201 clones annotated to genes based on sequences obtained from a leafy spurge EST-database [36]. Gene abbreviations and descriptions of all putative homologous leafy spurge genes (Additional file 1: Table S1) were obtained from an *Arabidopsis* website (www.arabidopsis.org). The details of cDNA preparation and qRT-PCR parameters were described previously by Chao [37]. Briefly, the comparative CT method was used to determine changes in target gene expression in test samples relative to a control sample. Fold difference in gene expression of test vs. control sample is 2^-ΔΔCT^, where ΔΔC_T_ = ΔC_T,test_ - ΔC_T,control_. Here, ΔC_T,test_ is the C_T_ value of test sample normalized to the endogenous reference gene, and ΔC_T,control_ is the C_T_ value of the control normalized to the same endogenous reference gene. SYBR green chemistry was used to produce fluorescent signal, and three technical replicates were used per sample for the qRT-PCR experiments. The C_T_ value of each gene is the average of three technique replicates. A leafy spurge SAND family gene was used as a reference; this gene was verified to be stably expressed during seed and bud development [38]. Values from three biological replicates were averaged, and data from 1d C seeds and endodormant buds were used for baseline expression. QbasePLUS version 2.4 software (Biogazelle, Ghent, Belgium) was used to normalize expression values and to perform statistical analyses. The difference in gene expression is designated as log2 and fold value (see Additional file 3: Table S3 for these two values).

### Cluster analysis and *t*-test

Transcript expression intensities were log2 transformed, and normalized with SAND family gene. Cluster analysis is done to group expression similarities of 201 genes in different phases of bud and seed samples. Euclidean distance (linear scaled) method and UPGMA clustering algorithm were used in this analysis. To identify genes with significant differential expression between two different phases of dormancy, unpaired two-sample t-tests were performed and genes at a p-value < 0.1 are considered as statistically significant.

## Additional files

Additional file 1: Table S1. Gene abbreviations/descriptions and primer pair sequences. Additional file 2: Table S2. Melting point temperatures and DNA bands for 201 amplicons. Additional file 3: Table S3. Differentially-expressed genes within buds and seeds for the 201 genes by qRT-PCR.
